# Semi-supervised multi-label collective classification ensemble for functional genomics

**DOI:** 10.1186/1471-2164-15-S9-S17

**Published:** 2014-12-08

**Authors:** Qingyao Wu, Yunming Ye, Shen-Shyang Ho, Shuigeng Zhou

**Affiliations:** 1 Shenzhen Key Laboratory of Internet Information Collaboration, Shenzhen Graduate School, Harbin Institute of Technology, Shenzhen, China; 2 School of Computer Engineering, Nanyang Technological University, Singapore; 3 Shanghai Key Lab of Intelligent Information Processing, and School of Computer Science, Fudan University, Shanghai, China

**Keywords:** Protein function prediction, protein interaction networks, collective classification, semi-supervised learning, multi-label learning

## Abstract

**Background:**

With the rapid accumulation of proteomic and genomic datasets in terms of genome-scale features and interaction networks through high-throughput experimental techniques, the process of manual predicting functional properties of the proteins has become increasingly cumbersome, and computational methods to automate this annotation task are urgently needed. Most of the approaches in predicting functional properties of proteins require to either identify a reliable set of labeled proteins with similar attribute features to unannotated proteins, or to learn from a fully-labeled protein interaction network with a large amount of labeled data. However, acquiring such labels can be very difficult in practice, especially for multi-label protein function prediction problems. Learning with only a few labeled data can lead to poor performance as limited supervision knowledge can be obtained from similar proteins or from connections between them. To effectively annotate proteins even in the paucity of labeled data, it is important to take advantage of all data sources that are available in this problem setting, including interaction networks, attribute feature information, correlations of functional labels, and unlabeled data.

**Results:**

In this paper, we show that the underlying nature of predicting functional properties of proteins using various data sources of relational data is a typical collective classification (CC) problem in machine learning. The protein functional prediction task with limited annotation is then cast into a semi-supervised multi-label collective classification (SMCC) framework. As such, we propose a novel generative model based SMCC algorithm, called GM-SMCC, to effectively compute the label probability distributions of unannotated protein instances and predict their functional properties. To further boost the predicting performance, we extend the method in an ensemble manner, called EGM-SMCC, by utilizing multiple heterogeneous networks with various latent linkages constructed to explicitly model the relationships among the nodes for effectively propagate the supervision knowledge from labeled to unlabeled nodes.

**Conclusion:**

Experimental results on a yeast gene dataset predicting the functions and localization of proteins demonstrate the effectiveness of the proposed method. In the comparison, we find that the performances of the proposed algorithms are better than the other compared algorithms.

## Background

Advances in biotechnology have enabled high-throughput experiments to generate a wide variety of genomic and proteomic data sources, including genome sequences, protein structure, and protein-protein interaction (PPI) networks.

Each data source provides a comprehensive view of the underlying mechanisms, and is represented as a set of features in a feature space or viewed as a graph structure where each individual is considered as a node. In the field of functional genomics, the process of manual annotation has become increasingly cumbersome with the rapid accumulation of the proteomic and genomic datasets. Computational methods to automate this task are urgently needed. Therefore, various computational methods have been proposed to automatically infer the functional properties of proteins using various data sources available (see [1] for a review).

Previous research in protein (or gene) function prediction can be partition into two classes of methods (feature-based approaches and graph-based approaches) according to the terms of input data and methodology. Feature-based machine learning algorithms require the instances to have a fixed set of attribute values from a feature space. The approaches involve extraction of features to encode the desired properties of a protein, and construction of a machine learning model for functional properties prediction. Some of the popularly used features are characteristics from amino acid sequence, textual repositories like MEDLINE, and more biologically meaningful features such as motifs derived from motif analysis of protein sequences, the isoelectric point and post-translational modifications. Via these constructed attribute features, a predictive model is learnt by training a classifier using annotated proteins, and then utilize this model to predict the functions of the proteins [2-5].

On the other hand, graph-based approaches use the network structure information to exploit proteins (or genes) sharing similar functional properties. Protein interaction networks are becoming increasingly rich and useful in delineating the biological characteristics of proteins. A review of computational approaches that are being used to measure protein interactions can be found in [6]. For instance, the Pearson's correlation coefficient is used to measure pairwise similarity between gene expression profiles. Specifically, the protein-protein interaction data can be modeled as a graph by considering individual proteins as the nodes, and the existence of an interaction between a pair of proteins as a link, graph-based or kernel-based classification algorithms are then used for protein data classification tasks based upon the protein interaction network [7-10].

Although many efforts have been made for automatically predicting functional properties of the proteins, this task still poses several significant challenges. First of all, existing feature-based methods and graph-based methods cannot guarantee good accuracy when there is only limited number of labeled data available. Most of the existing feature-based methods and graph-based methods require sufficiently large amount of labeled examples or a fully-labeled graph for training. However, acquiring such labels can be very expensive and time-consuming in practical applications. The performance of functional prediction might be degraded when the requirement of sufficient labeled data is not met. Furthermore, proteins are generally involved in more than one biological process, and thus they are annotated with multiple functions. Thus, it increases the difficulties of functional prediction. A promising idea to tackle these challenges (label deficiency and multiple function prediction problems) is to take advantage of multiple data sources and multiple functions of proteins for enhancing the prediction performance. To this end, we propose effective approaches that utilize all data sources that are available in this problem setting, including interaction networks, protein attribute features, label correlations, and unlabeled data for enhancing the performance of predicting functional properties of the proteins.

In this paper, we first show that the learning task underlying the protein function prediction using various data sources of relational data matches well with the collective classification [11-13] framework. Then, we propose a new generative model based semi-supervised multi-label collective classification algorithm, called GM-SMCC, for predicting proteins with multiple functions utilizing both labeled and unlabeled data in the learning process. To further boost the learning performance, we extend our proposed GM-SMCC method in an ensemble manner by constructing multiple latent networks. This approach, called ensemble of GM-SMCC model (EGM-SMCC), constructs various kinds of latent networks with various latent linkages to explicitly model the relationships among the node. We show how to effectively integrate these latent networks in an ensemble framework to improve the performance of protein function prediction.

We study the KDD Cup 2001 tasks of predicting functional properties (protein localization and their biological functions) of the protein corresponding to a given yeast gene. Experimental results show that the proposed algorithms (GM-SMCC and EGM-SMCC) can lead to performance superior to other compared feature-based approaches, graph-based approaches, and collective classification algorithms. In summary, the main contributions of this paper are listed as the following:

1. This article is the first one to examine the CC algorithm for protein function prediction using semi-supervised learning and multi-label learning techniques to leverage the unlabeled portion of the data and label correlation information in the partially-labeled PPI network, which only has limited number of annotations.

2. The proposed GM-SMCC algorithm is able to utilize various data sources for protein function prediction, where the instance features and interactions, as well as the label correlations can be naturally and explicitly exploited to predict a set of functional labels for an unannotated protein.

3. The proposed EGM-SMCC algorithm is a multi-network learning method which integrates multiple constructed latent graphs for protein function prediction using an ensemble framework. Via the multiple latent graphs constructed, the supervised knowledge can be propagated from labeled to unlabeled nodes effectively to boost the prediction performance.

## Prediction task formalization

The protein functional properties prediction task has been widely explored in the literature. An extensive review on this task is found in [1]. The approaches of protein function prediction can be categorized into two categories, feature-based methods and graph-based methods, in terms of input data and methodology.

**Feature-based methods**. For these methods, each protein is characterized as a feature vector **x***_i _*=<*f*_1_*, ..., f_d _*> with a fixed set of feature values. The feature vectors of the data then taken as input to machine learning algorithms to infer annotation rules for predicting unannotated proteins [14]. Learning algorithms that have been used include SVM [3], neural networks [15], random forest [16], and cotraining [14], to name a few. Typically, feature extraction is involved to extract desired features to represent information of proteins. Then a feature selection is used in the learning process to select the most useful features to train a classifier. A protein usually performs multiple functions. As such, several approaches handle the prediction problem using the multi-label learning framework. For instance, Barutcuoglu et al. [17] learn SVM classification model for predicting functions in the Gene Ontology using a hierarchical multi-label structure. Pandey et al. [18] incorporate function correlation for predicting protein functions using a weighted multi-label *k*NN classifier. Schietgat et al. [19] predict gene function using hierarchical multi-label decision tree ensembles.

**Graph-based methods**. These methods study protein function in the context of a network. The recent availability of protein interaction networks has spurred on the development of computational methods for analyzing such data in order to elucidate the relationships between protein interactions and functional properties. Sharan et al. [9] categorize the methods into two groups: direct annotation schemes, which infer the function of a protein based on its connections in the network; and module-assisted schemes which first identify modules of related proteins and then annotate each module based on the known functions of its members. Examples of direct annotation algorithms include neighborhood counting [8], graph theoretic methods [20], and Markov random field [21]. On the other hand, the model-assisted methods differ mainly in their module (or cluster) detection techniques. Examples of model detection methods include hierarchical clustering-based methods [22] and graph clustering-based methods [23]. Graph-based approaches using multi-label learning framework for prediction have also been studied [24-26].

Although a broad variety of interesting approaches have been developed, most of the methods mainly study the scenario where sufficient labeled data are available in the dataset. In this case, the supervision knowledge can be effectively used in the feature-based models and graph-based methods to achieve good learning performance. However, such labels are difficult and time-consuming to obtain. In sparse-labeled networks, one has only limited number of labeled nodes, say fewer than 10%, 5% or even 1%. The performance of prediction might be degraded due to the lack of annotated proteins [27]. It is thus natural to consider using various data sources of the protein data (including labeled and unlabeled) to improve the prediction performance.

**Collective classification**. The task of protein function prediction can be cast into the collective classification problem of building a predictive model from networked data. Generally, networked data can be represented by nodes (instances) interconnected with each other by edges reflecting the relation or dependence between the nodes. Information on the nodes is provided as a set of attribute features (e.g., words present in the web page). The class membership of an instance may influence the class membership of a related instance.

Conventional supervised learning methods assume that the instances to be classified are independent of each other, while *collective classification *jointly classifies interrelated instances by exploiting the interrelations among the instances [28,29]. For example, consider the task of predicting the topics of hyperlinked web pages. Conventional supervised learning approaches only use the attribute features derived from the content of the pages to classify each page. In contrast, collective classification methods use the link structure to construct additional relational features based on the labels of neighboring pages. We can count the number of different labels of the neighboring pages that are linked to each page as the relational features. Collective classification methods would then explicitly use the attribute features and the relational features together for classification.

Formally, the collective classification task is described as follows: Let *G *= (*V, E, X, Y, C*) be a graph dataset. *V *is a set of nodes {*v*_1_*, . . . , v_N _*}. *E *is the adjacency matrix where *E*(*i, j*) = 1 if node *v_i _*and node *v_j _*are connected and *E*(*i, j*) = 0 otherwise. *X *⊂ R*^d ^*consists of *d* dimensional vector instances. Each *x_i _*∈ *X *is an attribute vector for a node *v_i _*∈ *V . C *= {*c*_1_*, c*_2_*, ..., c_K_*} is the set of *K *possible labels. *Y *contains the set of label set *Y_i _*corresponding to instance *x_i _*for *i *= 1*, . . . , N *. Each *Y_i _*= [*Y_i_*_,1_, . . . , *Y_i,l_, . . . , Y_i,K _*] ∈ {0, 1}^k ^such that *Y_i,l _*= 1 means that *x_i _*is associated with *l *and *Y_i,l _*= 0 otherwise. We assume that we have $n^{\prime }$ label data ${\left\{\left(x_{i},Y_{i}\right)\right\}}_{i=1}^{n^{\prime }}$ and $n^{\prime \prime }$ unlabeled data ${\left\{\left(x_{i}\right)\right\}}_{i=n^{\prime }+1}^{n^{\prime }+n^{\prime \prime }}$ with $N=n^{\prime }+n^{\prime \prime }$. The task is to construct a function to predict the class label of unlabeled nodes using the labeled nodes in the graph.

When there are only limited number of labeled nodes in the task of predicting functional properties of proteins, i.e. $n^{\prime }\ll n^{\prime \prime }$, most of the proteins may not connect to labeled ones, which makes the task very challenging. As such, it is natural to consider some sort of semi-supervised learning. In the setting of semi-supervised learning, one utilizes both labeled and unlabeled data together to improve the performance [30].

## Methods

In this section, we present the (GM-SMCC) algorithm to address the task of predicting functional properties of proteins. Our approach is to model the problem as a generative model process to learn a probabilistic interpretation of the data for the estimation of the conditional distribution *p*(*c|x*) of the data, where *c *is a functional class and *x *is a protein instance.

### GM-SMCC

Given the dataset *X *= {*x*_1_*, ...,x_i_,..., x_N_} *with the attribute features *W *= {*w*_1_*,... ,w_j _,...,w_M_*}, we set up a generative model for the attribute features of the protein instances in *X *(including labeled and unlabeled data) and estimating the conditional distribution *P*(*c|x*) by using the pLSA model originally developed for latent topic analysis. Unlike other topic model based on latent topics, we adopt protein functional class *c_k _*as latent variables in the pLSA model and fixing *p*(*c_k_|x_i_*) for the annotated proteins in the learning process. The model is given as

$$P\left(x_{i},w_{j}\right)=P\left(x_{i}\right)\overset{K}{\underset{k=1}{\sum }}P\left(w_{j}|c_{k}\right)P\left(c_{k}|x_{i}\right)$$

where *P*(*c_k_|x_i_*) and *P*(*w_j _|c_k_*) are the probabilities that a protein instance *x_i _*is associated with functional class *c_k _*and the probability that attribute feature *w_j _*occurs in a protein with class *c_k_*, respectively. For efficient optimization, we utilize the log-likelihood. The likelihood function is transformed into:

(1) $$L=\overset{N}{\underset{i=1}{\sum }}\overset{M}{\underset{j=1}{\sum }}n\left(x_{i},w_{j}\right)\text{log}\overset{K}{\underset{k=1}{\sum }}P\left(w_{j}|c_{k}\right)P\left(c_{k}|x_{i}\right)$$

where *n*(*x_i_,w_j_*) is the frequency of *w_j _*occurring in *x_i_*, and *N,M *are the number of proteins and attribute features, respectively.

We exploit the knowledge of network topological structure of the data for better estimation of the conditional probability *P*(*c|x*) based on the assumption that nearby nodes tend to have similar labels. The basic assumption is that if two nodes *x_i _*and *x_s _*are connected in the network, these nearby nodes tend to share similar class labels, i.e., the distance of their conditional distribution *P*(*c|x_i_*) and *P*(*c|x_s_*) should be similar to each other. Here, we consider the Kullback-Leibler (KL) divergence to measure the distance of two distributions. Suppose the distribution of *P*(*c_k_|x_i_*) with respect to different classes is represented as a vector **z***_i _*= [*P*(*c*_1_*|xi*)*, · · ·, P*(*c_K_|x_i_*)]*^T ^*. Then the KLdivergence between **z***i *and **z***s *is defined as

$$D\left(z_{i}||z_{s}\right)=\overset{K}{\underset{k=1}{\sum }}P\left(c_{k}|x_{i}\right)\text{log}\frac{P\left(c_{k}|x_{i}\right)}{P\left(c_{k}|x_{s}\right)}$$

KL-divergence is not symmetric, and thus we use the following symmetric KL-divergence

$$D\left(z_{i},z_{s}\right)=\frac{1}{2}\left(D\left(z_{i}||z_{s}\right),)\right)+D\left(D\left(z_{s}||z_{i}\right)\right)$$

to measure the distance of two distributions. Here,*D*(**z***_i_; ***z***_s_*) is always nonnegative.

As discussed above, our idea is to smooth the distribution *P*(*c|x*) over the network. If two proteins are connected with interactions, then their conditional distributions *P*(*c|x_i_*) and *P*(*c|x_s_*) should be close to each other. Such local smoothness in terms of the network topology is explicitly incorporated into the generative model through a network regularizer

(2) $$R=\overset{N}{\underset{i,s=1}{\sum }}\left(D\left(z_{i},z_{s}\right)\right)E_{is}$$

where *E *is the adjacency matrix to represent the network topology, *E_i,s _*= 1 if *v_i _*and *v_s _*are connected, and *E_i,s _*= 0 otherwise.

In protein functional properties prediction, proteins generally involve multiple biological processes and have multiple functions. Thus, it is crucial to take the label correlations into account to better predict their functional classes. Here, we further generalized the generative model to support this general setting. Recall that the network regularizer  R is used to smooth label probability distribution over the intrinsic network structure. One hopes that the resulting distribution is able to be smoothed with respect to the class label correlations. A natural assumption here could be that if two class labels *c_k _*and *c_l _*are related, then the distribution *P*(*c_k_|x_i_*) and *P*(*c_l_|x_i_*) with respect to different instances should be also similar to each other.

In particular, we construct a label-to-label affinity graph with *K *vertices where each vertex corresponds to one class label. For each pairwise vertices, we put edges between them and compute their weighting. There are many choices to define the weight matrix **F **= [*F_kl_*] on the affinity graph. Specifically, we use the commonly used dot-product as follows

$$F_{kl}=Y_{k}^{T}Y_{l},$$

where *Y_k _*= [*Y*_1_*,_k_, · · ·, Y_N,k_*]*^T ^*is the label distribution over the instances, such that *Y_i,k _*is nonzero if *x_i _*belongs to class *c_k _*and the remaining elements are zero. Here, *Y_k _*is normalized to 1. The dot product of two vectors is equivalent to their cosine similarity.

Suppose the vector representation of *P*(*c_k_|x_i_*) with respect to different instances is **r***_k _*= [*P*(*c_k_|x*_1_)*, · · ·, P*(*c_k_|x_N_*)]*^T ^*.

we define the KL-divergence between **r***_k _*and **r***_l _*for pairwise class labels as follows

$$\begin{array}{c} D\left(r_{k}||r_{l}\right)=\overset{N}{\underset{i=1}{\sum }}P\left(c_{k}|x_{i}\right)\text{log}\frac{P\left(c_{k}|x_{i}\right)}{P\left(c_{l}|x_{i}\right)}\\ D\left(r_{k},r_{l}\right)=\frac{1}{2}\left(D\left(r_{k}||r_{l}\right),)\right)+D\left(D\left(r_{l}||r_{k}\right)\right) \end{array}$$

By using the label affinity matrix **F **and the symmetric KL-divergence defined above, we defined the label regularizer

(3) $$H=\overset{K}{\underset{k,l=1}{\sum }}\left(D\left(r_{k},r_{l}\right)\right)F_{k,l}$$

to smooth the distribution *P *(*c*|*x*).

Incorporating the smoothness terms (2) and (3) into the objective function in (1), we have the following new objective function

$$\begin{array}{c} O=L-\alpha R-\beta H\\ \;=\overset{N}{\underset{i=1}{\sum }}\overset{M}{\underset{j=1}{\sum }}n\left(x_{i},w_{j}\right)\text{log}\overset{K}{\underset{k=1}{\sum }}P\left(w_{j}|c_{k}\right)P\left(c_{k}|x_{i}\right)\\ \;-\frac{\alpha }{2}\overset{N}{\underset{i,s=1}{\sum }}\overset{K}{\underset{k=1}{\sum }}\left(P\left(c_{k}|x_{i}\right)\text{log}\frac{P\left(c_{k}|x_{i}\right)}{P\left(c_{k}|x_{s}\right)}+P\left(c_{k}|x_{s}\right)\text{log}\frac{P\left(c_{k}|x_{s}\right)}{P\left(c_{k}|x_{i}\right)}\right)E_{is}\\ \;-\frac{\beta }{2}\overset{N}{\underset{i=1}{\sum }}\overset{K}{\underset{k,l=1}{\sum }}\left(P\left(c_{k}|x_{i}\right)\text{log}\frac{P\left(c_{k}|x_{i}\right)}{P\left(c_{l}|x_{i}\right)}+P\left(c_{l}|x_{i}\right)\text{log}\frac{P\left(c_{l}|x_{i}\right)}{P\left(c_{k}|x_{i}\right)}\right)F_{kl} \end{array}$$

where *α *and *β *are the regularization parameters. When *α *= 0 and *β *= 0, maximizing  O is equivalent to performing learning using the original pLSA model.

For the annotated proteins, their probability distributions *P *(*c*|*x*) are fixed in the learning process. Specifically, the probability assignments are defined as a uniform distribution based on the known functional class labels as follows

(5) $$P\left(c_{k}|x_{i}\right)=\left\{\begin{array}{cc} 1/l_{x} & \text{if}\;\;\;x_{i}\text{is}\;\;\;\text{labeled}\;\;c_{k}\\ 0 & \text{otherwise} \end{array}\right)$$

where *lx *is the number of functional classes for an annotated protein *xi*.

For the unannotated proteins, we maximize the log-likelihood function  O to compute their probabilistic distributions. The resulting probability distribution *P *(*c*|*x_i_*) with respect to a given instance *xi *indicates the importance of a set of functions to the protein. One hopes that the *P *(*c_l_*|*x_i_*) of the relevant labels are close to each other, and their values should be larger than those of the irrelevant labels. Hence, to make prediction of *x_i_*, we first rank the labels according to *P *(*c_k _*|*x_i_*). Then we separate the set of labels into relevant and irrelevant label subsets according to the largest change observed across the sorted *P *(*c_k _*|*x_i_*). That is, we seek the largest change between two successive *P *(*c_k _*|*x_i_*) and *P *(*c_k_*_+1_|*x_i_*) in terms of their sorted orders. Their median value, say *t *= (*P*(*c_k _*|*x_i_*) + *P *(*c_k_*_+1_|*x_i_*))*/*2, is used as splitting threshold to separate the class labels into relevant set and irrelevant set, where the the relevant set consists of the labels with probabilities larger than the threshold *t*, and the irrelevant set contains the remaining labels.

### Model fitting with the EM algorithm

Our proposed approach, GM-SMCC, utilizes the generative model with both network and label regularization for protein function prediction, and parameter estimation is different from original PLSA [31] or previous work utilizing PLSA with manifold learning for unsupervised data clustering [32]. Next, we introduce the EM algorithm used in the proposed GM-SMCC approach for finding maximum likelihood parameter estimates.

In the proposed generative model, we have *N K *+ *M K *parameters {*P *(*w_j _*|*c_k _*)*, P *(*c_k _*|*x_i_*)} where the class labels *ck *are considered as the latent variables. For convenience, we denote these parameters as Θ. We use the EM algorithm which alternates between an expectation step (E-step) and a maximization step (M-step) to estimate the parameters in the proposed GM-SMCC model.

### E-step

The E-step is the same as in the pLSA model. The posterior probabilities for the latent variables *P *(*c_k_*|*x_i_, w_j_*) is computed as follows

(6) $$P\left(c_{k}|x_{i},w_{j}\right)=\frac{P\left(w_{j}|c_{k}\right)P\left(c_{k}|x_{i}\right)}{\sum_{l=1}^{K}P\left(w_{j}|c_{l}\right)P\left(c_{l}|x_{i}\right)}$$

### M-step

The M-step re-estimation for {*P *(*w_j _*|*c_k _*)} is the same as that in the pLSA model as follows

(7) $$P\left(w_{j}|c_{k}\right)=\frac{\sum_{i=1}^{N}n\left(x_{i},w_{j}\right)P\left(c_{k}|x_{i},w_{j}\right)}{\sum_{m=1}^{M}\sum_{i=1}^{N}n\left(x_{i},w_{m}\right)P\left(c_{k}|x_{i},w_{m}\right)}$$

In the M-step, parameters are updated based on the expected complete data log-likelihood which depends on the posterior probabilities computed in the E-step [31]. The expected complete data log-likelihood of (4) is given by

$$\begin{array}{c} Q\left(\Theta \right)=Q_{1}\left(\Theta \right)+Q_{2}\left(\Theta \right)\\ \;=\overset{N}{\underset{i=1}{\sum }}\overset{M}{\underset{j=1}{\sum }}n\left(x_{i},y_{j}\right)\overset{K}{\underset{k=1}{\sum }}P\left(c_{k}|x_{i},w_{j}\right)\text{log}\left[P\left(w_{j}|c_{k}\right)P\left(c_{k}|x_{i}\right)\right]\\ \;-\alpha \overset{N}{\underset{i,s=1}{\sum }}\overset{K}{\underset{k,l=1}{\sum }}D\left(P_{i}\left(c_{k}\right),P_{s}\left(c_{k}\right)\right)E_{is}\\ \;-\beta \overset{N}{\underset{i,s=1}{\sum }}\overset{K}{\underset{k,l=1}{\sum }}D\left(P_{i}\left(c_{k}\right),P_{i}\left(c_{l}\right)\right)F_{kl} \end{array}$$

using the posterior probabilities computed in the E-step.

We need to maximize $Q\left(\Theta \right)$ with respect to the parameter Θ subject to the constraints $\sum_{k=1}^{K}P\left(c_{k}|x_{i}\right)=1$ and $\sum_{j=1}^{M}P\left(w_{j}|c_{k}\right)=1$. Therefore, we augment $Q\left(\Theta \right)$ by the appropriate Lagrange multipliers *ρ_i _*to obtain

(8) $$Q^{\prime }=Q\left(\Theta \right)+\overset{N}{\underset{i=1}{\sum }}\rho_{i}\left(1-\overset{K}{\underset{k=1}{\sum }}P\left(c_{k}|x_{i}\right)\right)$$

Maximization of $Q^{\prime }$ with respect to *P *(*c_k _*|*x_i_*) leads to the following set of equations:

(9) $$\frac{\sum_{j=1}^{M}n\left(x_{i},w_{j}\right)P\left(c_{k}|x_{i},w_{j}\right)}{P\left(c_{k}|x_{i}\right)}-\rho_{i}$$

$$\begin{array}{c} -\frac{\alpha }{2}\overset{N}{\underset{s=1}{\sum }}\left(\text{log}\frac{P\left(c_{k}|x_{i}\right)}{P\left(c_{k}|x_{s}\right)}+1-\frac{P\left(c_{k}|x_{s}\right)}{P\left(c_{k}|x_{i}\right)}\right)E_{is}\\ -\frac{\beta }{2}\overset{K}{\underset{l=1}{\sum }}\left(\text{log}\frac{P\left(c_{k}|x_{i}\right)}{P\left(c_{l}|x_{i}\right)}+1-\frac{P\left(c_{l}|x_{i}\right)}{P\left(c_{k}|x_{i}\right)}\right)F_{kl}=0 \end{array}$$

where 1 ≤ *i *≤ *N*, 1 ≤ *k *≤ *K*.

We expect that if the attribute features of two proteins *x_i _*and *x_s _*are close (i.e., *E_is _*is large), then the distribution *P *(*c_k _*|*x_i_*) and *P *(*c_k _*|*x_s_*) are similar to each other, i.e., *P*(*c_k _*|*x_i_*) will be close to *P *(*c_k _*|*x_s_*). We have

$${\left(\frac{P\left(c_{k}|x_{i}\right)}{P\left(c_{k}|x_{s}\right)}\right)}^{E_{is}}\approx 1$$

Similarly, if two functions *c_k _*and *c_l _*are close (i.e., *F_kl _*is large), then the distribution *P *(*c_k _*|*x_i_*) and *P *(*c_l_*|*x_i_*) are similar to each other, i.e., *P *(*c_k _*|*x_i_*) will be close to *P *(*c_l_*|*x_i_*).

$${\left(\frac{P\left(c_{k}|x_{i}\right)}{P\left(c_{l}|x_{i}\right)}\right)}^{F_{kl}}\approx 1$$

We have,

By using the approximation

$$\text{log}\left(x\right)\approx 1-\frac{1}{x},x\to 1,$$

(9) can be written as

(10) $$\begin{array}{c} \frac{\sum_{j=1}^{M}n\left(x_{i},w_{j}\right)P\left(c_{k}|x_{i},w_{j}\right)}{P\left(c_{k}|x_{i}\right)}\\ \;-\rho_{i}-\frac{1}{P\left(c_{k}|x_{i}\right)}\left(\alpha A_{1}+\beta A_{2}\right)=0 \end{array}$$

where 1 ≤ *i *≤ *N*, 1 ≤ *k *≤ *K*,

$$\begin{array}{c} A_{1}=\overset{N}{\underset{s=1}{\sum }}\left(P\left(c_{k}|x_{i}\right)-P\left(c_{k}|x_{s}\right)\right)E_{is}\\ \;=P\left(c_{k}|x_{i}\right)\overset{N}{\underset{s=1}{\sum }}E_{is}-\overset{N}{\underset{s=1}{\sum }}P\left(c_{k}|x_{s}\right)E_{is} \end{array}$$

and

$$\begin{array}{c} A_{2}=\overset{K}{\underset{l=1}{\sum }}\left(P\left(c_{k}|x_{i}\right)-P\left(c_{l}|x_{i}\right)\right)F_{kl}\\ \;=P\left(c_{k}|x_{i}\right)\overset{K}{\underset{l=1}{\sum }}F_{kl}-\overset{K}{\underset{l=1}{\sum }}P\left(c_{l}|x_{i}\right)F_{kl} \end{array}$$

To obtain the M-step re-estimation for *P *(*c*|*x*), we construct six *N K*-by-*N K *matrices: **Z**, **Ω**, **D**, **B**, **U**, and **R**.

First, we construct a *K*-by-*K *block diagonal matrix **D **= [**D***_i,j_*] based on the adjacency matrix *E*, where the (*i, j*)th block of **D **is a *N *-by-*N *matrix **D***_i,j _*= [*d_i,j,s,t_*]*s,t*=1*,...,N *. All the entries of **D **are equal to 0 except the diagonal entries $d_{i,i.s.s}=\underset{s}{\sum }E_{is}$

Next, we construct another *K*-by-*K *block diagonal matrix **B **= [**B***_i,j_*] where its (*i, j*)th block is also a *N *-by-*N *matrix **B***_i,j _*= [*b_i,j,s,t_*]*s,t*=1*,...,N *. The entries of **B **are equal to 0 when *i *≠ *j*; otherwise, if *i *= *j*, then we have *b_i,j,s,t _*= *E_st_*.

Then, we construct a *N *-by-*N *block diagonal matrix **U **= [**U***_i,j_*] based on the label correlation matrix *F *, where the (*i, j*)th block of **U **is a *K*-by-*K *matrix **U***_i,i _*= [*u_i,i,s,t_*]*_s_,t*=1*,...,K *. All non-diagonal entries of **U **are equal to 0 and the diagonal entries $u_{i,i.s.s}=\underset{s}{\sum }F_{sl}$.

The matrix **R **= [**R***_i,j_*] is another *N *-by-*N *block matrix where its (*i, j*)th block is a *K*-by-*K *matrix **R***_i,j _*= [*r_i,j,s,t_*]*_s_,t*=1*,...,N *. Indeed, each **R***_i,j_*, for *i, j *= 1*, ..., K*, is a diagonal matrix *r_i,j,s,s _*= *F_ij _*.

The matrix **Z **is a *K*-by-1 block vector, where its *k*-th entry **Z***_k _*is a *N *dimensional vector defined as follows

$$\begin{array}{c} Z_{k}=\left[\begin{array}{c} \sum_{j=1}^{M}n\left(x_{1},w_{j}\right)P\left(c_{k}|x_{1},w_{j}\right)\\ \cdots \\ \sum_{j=1}^{M}n\left(x_{N},w_{j}\right)P\left(c_{k}|x_{N},w_{j}\right) \end{array}\right] \end{array}$$

The matrix **Ω **is a *K*-by-*K *block matrix where its (*i, j*)th block is a *N *-by-*N *diagonal matrix. All the non-diagonal entries of **Ω **are equal to 0 and the diagonal entries

$$\Omega_{i,i,s,s}=\rho_{i}=\sum_{k=1}^{K}\sum_{j=1}^{M}n\left(x_{s},w_{j}\right)P\left(c_{k}|x_{s},w_{j}\right)$$

Let **y **denotes a *K*-by-1 block matrix where

$$Y_{k}={\left[P\left(c_{k}|x_{1}\right),\cdots \;,P\left(c_{k}|x_{N}\right)\right]}^{T}$$

The system of equations in (9) is approximated using (10) and can be solved using the following matrix form:

(11) $$\text{Z}-\Omega y-\alpha \left(\text{D}-\text{B}\right)y-\beta \left(\text{U}-\text{R}\right)y=0$$

Thus, the M-step re-estimation for *P *(*c*|*x*) is

(12) $$y={\left(\Omega +\alpha \left(\text{D}-\text{B}\right)+\beta \left(\text{U}-\text{R}\right)\right)}^{-1}\text{Z}$$

The E-step (6) and M-steps (7) and (12) are alternated until the objective function (4) converges.

In the initialization step of the EM algorithm, the values of *P *(*w_i_*|*c_k _*) and *P *(*c_k _*|*x_i_*) are initialized based on the class priors according to the annotated proteins. We assume that each feature *w_j _*is conditionally independent to each other given the label *c_k _*. Concretely, *P *(*w_j_*|*c_k _*) are initialized as $P\left(w_{j}|c_{k}\right)=\frac{n\left(w_{j},c_{k}\right)}{\sum_{i}n\left(w_{i},c_{k}\right)}$, where *n*(*w_j _, c_k _*) is the frequency of *w_j _*and *c_k _*co-occuring. The label distribution *P *(*c_k _*|*x_i_*) for unannotated proteins are initialized as $P\left(c_{k}|x_{i}\right)=\frac{\sum_{i}n\left(c_{k},x_{i}\right)}{\sum_{l}n_{i}\left(c_{l},x_{i}\right)}$, where *n*(*c_k _, x_i_*) = 1 if *x_i _*is associated with *c_k _*and 0 otherwise. In each iteration of the EM algorithm, the probability assignments of *P *(*c*|*x*) for labeled data are reset according to the known functional class labels as in Eq. (5).

### EGM-SMCC algorithm

The power of the network regularizer in Eq. (4) of our proposed GM-SMCC model lies in the fact that the linkages of the network generally exhibit predictable relationships between class labels of linked proteins. Suppose we have an unannotated protein, and we have a good understanding of the relationship between the functions of this protein and the functional properties of its labeled neighbors, then we should be able to make a good prediction of the protein functional properties based on the linkage information.

In the proposed GM-SMCC model, we use the autocorrelation in the protein interaction network which may provide some inconsistent linkages between the proteins not sharing similar functional properties. In the studies of functional genomics, if more information is available, one can derive more effective networks for capturing useful relationships between the proteins to propagate the supervision knowledge from labeled nodes to unlabeled nodes.

In the real-world, protein data are associated with various data sources. For example, the proteins are associated with attribute features; those proteins with similar feature values may also be similar in their associated functions. Also, the proteins are associated with a set of functional labels, which can be represented by label features that are useful for evaluating the pairwise similarity of protein instances. These latent linkages are already embedded in the data. We can exploit this knowledge to construct the latent graphs for more effective label prediction.

In this paper, in addition to the PPI network, we introduce two types of latent linkages to construct latent graphs. Based on the latent graphs we constructed, we extend our proposed generative model in an ensemble manner to further boost the prediction performance.

Given the adjacency matrices ${\left\{E^{\left(i\right)}\right\}}_{i=1}^{q}$ of *q *latent graphs, the proposed ensemble algorithm, namely EGM-SMCC, is described in Algorithm 1. In the EGM-SMCC algorithm, we learn an individual GM-SMCC model on each of the constructed latent graph, and then combine the learned models to obtain a more reliable prediction than that of the model on a single latent graph.

**Algorithm 1 **EGM-SMCC

**Input: **${\left\{E^{\left(i\right)}\right\}}_{i=1}^{q}$, *X, Y *, the parameters *α *and *β*

Output: y

Procedure:

1: **for ***i *= 1 to *q ***do**

2:    Learn a GM-SMCC model using the constructed latent graph *E*^(*i*)^. In the GM-SMCC model, compute the network regularizer  R in Eq. (2) according to *E*^(*i*)^;

3:    Use EM algorithm to optimize the GM-SMCC model to compute the label probability distribution **y**^(i)^;

4: **end for**

5: Combine the results of *q *learned models **y**^(*i*)^, **y**^(*i*^),..., **y**^(*q*) ^into an ensemble prediction as $y=\frac{1}{q}\overset{q}{\underset{i=1}{\sum }}y^{\left(i\right)}$

**Figure 1:**
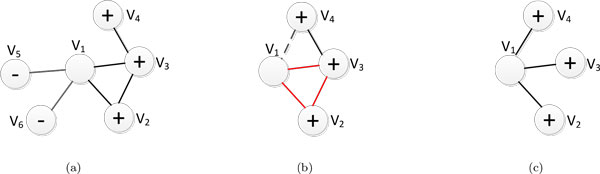
**An example of latent graphs used in the proposed EGM-SMCC model**. (a) PPI latent graph, i,e., the original interaction network, where the ground-truth label of the center node *v*_1 _is "+", but it is difficult to predict the label (+ or -) for node *v*_1 _since it has the same number of positive and negative neighboring nodes; (b) even-step random walk latent graph, the directed neighbors with the same label ("+") to *v*_1 _have triangle edges (red lines), hence they are reachable from *v*_1 _using even-step random walks. On the other hand, the indirected neighbors (from the + nodes) in network are linked by creating edges (dash line) using even-step random walks; (c) prediction similarity latent graph using *k*NN graph construction. In the *k*NN graph, a node pair share an indirected edge if the two nodes are *k*-nearest neighbors. In the example, we set *k *= 3.

The basic idea of constructing latent graphs is to link together the protein nodes, such that nodes which are closer in the graphs will tend to have the same functional labels, and the nodes which are disconnected will tend to have different functional labels. Via the latent linkages in the latent graphs we constructed, knowledge from labeled nodes can be propagated to unlabeled nodes more effectively, such as the example in Figure 1. Next, we introduce three type of latent linkages to construct latent graphs that can be easily computed from the data. For each individual latent graph, we compute a weight *E_ij _*for each entry of its adjacency matrix where *E_i,j _*is large indicates two nodes are close together, and vice versa.

**PPI latent graph: **In our ensemble model, we consider the PPI network as a latent graph, and construct the adjacency matrix *E*_(1) _of the PPI latent graph as follows

$$E_{ij}^{\left(1\right)}=E\left(i,j\right)$$

where *E*(*i, j*) = 1 if node *v_i _*and node *v_j _*are connected in the PPI network, and *E*(*i, j*) = 0 otherwise.

**Random walk latent graph: **When the underlying autocorrelation of original PPI network is small, i.e., some connected nodes may not share the same class label, the learning method based on the original PPI network might be affected.

It is observed that proteins that interact with level-2 neighbors (indirect neighbors in the PPI network) also have a great likelihood of sharing similar characteristics [8]. To this end, we use the idea of *even-step *random walk with restart (ERWR) [33] to compute the weights of the latent linkages. Intuitively, we assume that linkages to directed neighbors with the same function class with the target protein of interest typically have triangle structures (see Figure 1(b)). These neighbors (*v*_2 _and *v*_3_) are able to obtain high scores using ERWR because they are well-connected in the PPI network. On the other hand, ERWR can avoid the immediate neighbors (e.g., *v*_1 _and *v*_2_) with inconsistent linkages that negatively influence the predictions because they are sparsely-connected. ERWR can also exploit the indirect neighbor data by adding linkages to level-2 neighbors (e.g., *v*4) that are well-connected to level-1 neighbors.

Given the adjacency matrix *E *of the PPI network, we compute *P *= *EE *and normalize its entries with respect to each column to obtain a normalized transition probability matrix *P *. The ERWR random walker iteratively visits neighborhood nodes with transition probability given in *P *. Also at each step, it has probability *α *(e.g., *α *= 0.1) to return to the start node. We define the adjacency matrix *E*^(2) ^of the random walk latent graph as follows

$$E_{ij}^{\left(2\right)}=R\left(i,j\right)$$

where $R=\sum_{t=1}^{T}\alpha {\left(1-\alpha \right)}^{t}p^{t}$ is the steady-state probability matrix after *T *steps.

**Prediction similarity latent graph: **We also consider the values of class labels of the annotated proteins as input features to build a classifier that predicts all unlabeled proteins. Specifically, we use SVM classifier with probability outputs implemented in the LIBSVM library [34] to compute $Y_{i}={\left[P\left(c_{1}|x_{i}\right),P\left(c_{2}|x_{i}\right),\ldots ,P\left(c_{q}|x_{i}\right)\right]}^{T}$ such that *P *(*c_j _*|*x_i_*) is the confidence of a protein *x_i _*belongs to the class *c_j _*. The adjacency matrix *E*^(3) ^of latent graph based on the prediction confidences is defined as follows

$$E_{ij}^{\left(3\right)}=Y_{i}^{T}Y_{j}$$

Here, *Y_i _*and *Y_j _*are normalized to unit length, thus the dot product of the two vectors is equivalent to their cosine similarity.

In the prediction similarity latent graph, there are many entries being close to zero. It may not be necessary to consider these entries. Therefore, we use a *k*NN construction scheme for graph. We connect two nodes *v_i _*and *v_j _*if *v_j _*is among the *k*-nearest neighbors of *v_i _*or if *v_j _*is among the *k*-nearest neighbors of *v_i _*[35]. It is obvious that the number of edges is *O*(*N *) and the graph is symmetric. We define a sparse adjacency matrix for *k*NN graph as follows

$${Ê}_{i,j}^{\left(3\right)}=\left\{\begin{array}{cc} 1, & \text{if}\;\;v_{i}\in N_{k}\left(j\right)\;\;\;\text{or}\;\;\;v_{j}\in N_{k}\left(i\right)\\ 0, & \text{otherwise}\text{.} \end{array}\right)$$

where $N_{k}\left(i\right)$ is the set of *k *nearest neighbors of *v_i_*. In practice, we find that *k *does not need tuning. We use *k *= 10 nearest neighbors for each data set.

### Experiments

In this section, we discuss the extensive experimental results to compare the performance of our proposed methods with the other baselines: SVM, wvRN+RL, ICA, semi-ICA, and ICML, and show that the proposed methods are able to achieve better performance against these baselines.

### Yeast dataset and baselines

We conduct experiments to predict properties of the proteins corresponding to a given yeast gene from KDD Cup 2001 [36]. In particular, we formulated two prediction problems based on the properties of the proteins. Problem (1) is to predict the localization of the proteins encoded by the genes. It is a binary problem, i.e., a protein is localized (or not localized) to the corresponding organelle. Problem (2) is to predict the functions of the proteins, which a multi-label problem, i.e., a protein can have more than one function. There are totally 14 functional classes in the dataset.

The dataset for these two problems consisted 1,243 protein instances and 1,806 interactions among the pair of proteins interact with one another. The protein features include the attributes refer to the chromosome on which the genes appears, to whether the gene is essential for survival, observable characteristics of the phenotype, structural category of the protein, the existence of characteristic motifs in the amino acid sequence of the protein, and whether the protein forms larger proteins with others [36,14].

We evaluate the performance of problem (1) by classification accuracy, and problem (2) by three multi-label learning evaluation metrics, i.e., *Coverage, RankingLoss*, and *MacroF1 *[37]. These criteria are defined as follows

*Coverage *evaluates how far we need, on the average, to go down the list of labels in order to cover all the true labels of an instance:

$$\text{Coverage}\left(f\right)=\frac{1}{N}\overset{N}{\underset{i=1}{\sum }}\underset{c_{k}\in Y_{i}}{\text{max}}\;\;rank_{s}\left(x_{i},c_{k}\right)-1.$$

where *rank_s_*(*x_i_, c_k _*) denotes the ranks of class label *c_k _*de-rived from a confidence function *s*(*x_i_, c_k _*) which indicates the confidence for the class label *c_k _*to be a proper label of *x_i_*.

*Ranking loss *evaluates the average fraction of label pairsthat are reversely ordered for the instance:

$$Ranking\;\;\;Loss\;\;\left(f\right)=\frac{1}{N}\overset{N}{\underset{i=1}{\sum }}\frac{1}{\left|Y_{i}\right|\left|{Ȳ}_{i}\right|}\cdot \left|R_{i}\right|,$$

where $R_{i}=\left\{\left(c_{1},c_{2}\right)|h\left(x_{i},c_{1}\right)\leq h\left(x_{i},c_{2}\right),\left(c_{1},c_{2}\right)\in Y_{i}\times {Ȳ}_{i}\right\}$, and  Ȳ denotes the complementary set of *Y_i_*.

*MacroF1 *is the harmonic mean between precision and recall, where the average is calculated per label and then averaged across all labels. It is defined as

$$MacroF1=\frac{1}{K}\overset{K}{\underset{k=1}{\sum }}\frac{2\times p_{k}\times r_{k}}{p_{k}+r_{k}}$$

where *p_k _*and *r_k _*are the precision and recall of the *k*-th label.

To validate the performance of our proposed algorithms, we compare our approach with four baseline methods:

**1. SVM **[34]. This baseline is a feature-based method only using the attribute features of the proteins for learning without considering using any network information.

**2. wvRN+RL **[38]. This algorithm is a relational-only method using only the PPI network for prediction. wvRN+RL computes a new label distribution for an unlabeled node by averaging the current estimated distributions of its linked neighbors. This process is repeated until reaching the maximum iteration number.

**3. ICA **[28]. This denotes a collective classification algorithm which uses both attribute features and relational features to train a base classifier for prediction. The relational features are constructed based on the labels of neighbors. ICA uses an iterative process whereby the relational features are recomputed in each iteration until a fixed number of iterations is reached. Prior work has found logistic regression (LR) to be superior to other classifiers such as naive bayes and *k*NN, as base classifier for ICA. Therefore, we use LR as the local classifier for ICA in the experiments.

**4. semi-ICA **[39]. This method extends ICA to leverage the unlabeled data using semi-supervised learning. There are four semi-ICA variants (KNOWN-EM, ALL-EM, KNOWN-ONEPASS, ALL-ONEPASS) for semi-ICA, we run all four variants and choose the best one as the result of semi-ICA.

**5. ICML **[13]. This method extends ICA to handle multi-label learning by constructing additional label correlation features to exploit the dependencies among the labels as additional input features to learn base classifier. The ICML algorithm is also based on an iterative framework similar to ICA.

It is generally more difficult to determine the classifier parameter values when the number of labeled data available is smaller (which is the focus of this study). For the SVM classifier, we use the LibSVM [34] with linear kernel as base classifier, and simply set the penalty parameter *C *= 1.0 for the SVM as default. The maximum number of iterations for ICA, semi-ICA are set to 10, and we use logistic regression as their base classifier as in [39,13]. While the wvRN+RL uses 1000 iterations. The parameters *α *and *β *for our proposed method are set to 3 and 0.1. The parameter selection issue is discussed in the later section.

### Results on protein localization prediction

We first consider problem (1) of KDD Cup 2001, i.e., the protein localization prediction problem. We set *α ≠ *0 and *β *= 0 in our proposed method, and compare GM-SMCC with the learning algorithms: SVM, wvRN+RN, ICA and semi-ICA. The performance is measured in terms of classification accuracy.

**Figure 2:**
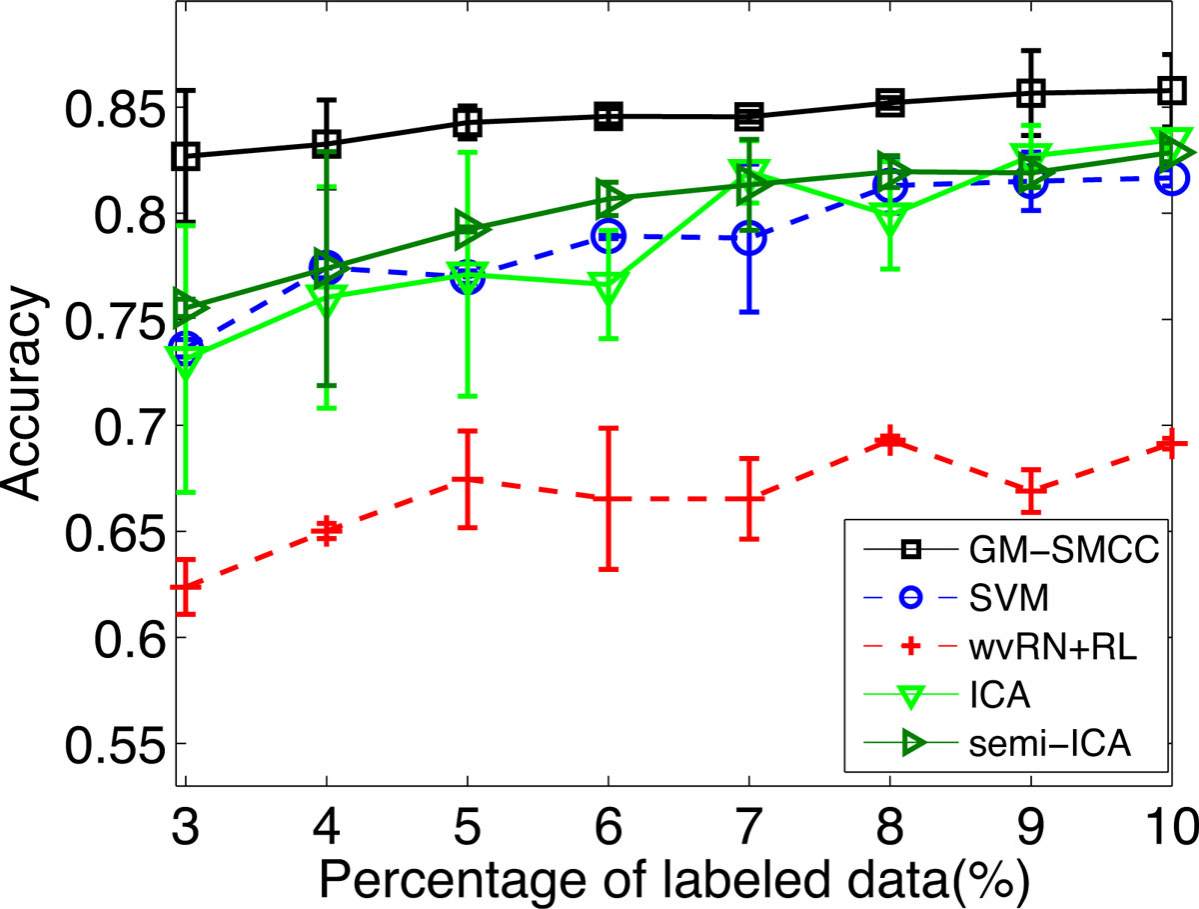
**Classification accuracy on problem (1) of KDD Cup 2001 dataset**.

We compare the performance of the comparison algorithms by varying the number of labeled data ranging from 3% to 10% with an interval of 1%. For each labeled/unlabeled data split, we execute an algorithm for 10 runs by randomly selecting data split, and report the performance (mean and standard deviation) over 10 runs for the algorithms. Figure 2 shows the experimental results. As we can see from the figure, the overall picture taken from the experiments is clearly in favor of our proposed GM-SMCC. The performance of GM-SMCC consistently outperforms the other algorithms across different percentages of labeled data. On average, the accuracy over different percentages for GM-SMCC, semi-ICA, ICA, SVM and wvRN+RL are 0.845, 0.801, 0.788, 0.788 and 0.666. GM-SMCC performs best followed by semi-ICA. The 3rd best methods are ICA and SVM. Their performances are comparable. The relational-only method wvRN+RL performs the worst.

We note that a smaller number of label data is the most interesting case for our algorithm because it is not reliable for prediction due to the inadequacy of supervised knowledge in the labeled dataset. Thus it is more desired that other data sources can be utilized together to improve the prediction performance. A closer examination of the results in Figure 2 show that the smaller the percentage of the labeled data is involved, the larger improvement GM-SMCC achieves. GM-SMCC achieves the largest improvement against 2nd best method when there are only 3% of labeled data (GM-SMCC: 0.82 versus semi-ICA: 0.75). We also conduct pairwise t-test at 0.05 significance level to assess the statistical significance of the differences in performance of GM-SMCC and the other test algorithms using 3% of labeled data. The performance of GM-SMCC is significant better than those of the other baseline methods. This result illustrates the advantages of our methods when there are an extremely small number of labeled data. This is consistent with our earlier assertions that our approach can work even in the paucity of annotated proteins by exploring various data sources, including interaction networks, attribute features, and unlabeled data.

In this study, three types of latent graphs are utilized (see the EGM-SMCC section). It is thus interesting to investigate the performance of GM-SMCC using a single latent graph, and the performance of EGM-SMCC utilizing multiple latent graphs. We test the performance of GM-SMCC and EGM-SMCC on the KDD Cup 2001 dataset with different label ratio from 3% to 10%. The experimental results are given in Table 1, where GM-SMCC-1, GM-SMCC-2 and GM-SMCC-3 denote the single-graph model using the PPI latent graph (*E*^(1)^), the random walk latent graph (*E*^(2)^) and the prediction similarity latent graph (*E*^(3)^), respectively. While GM-SMCC-mean denotes the single-graph model using a latent graph constructed by averaging the weighing values of *E*^(1)^, *E*^(2) ^and *E*^(3)^.

**Table 1:** Accuracy (mean*±*standard deviation) of GM-SMCC and EGM-SMCC against different label ratio on problem (1) of KDD Cup 2001.

label ratio	GM-SMCC-1	GM-SMCC-2	GM-SMCC-3	GM-SMCC-mean	EGM-SMCC
3%	0.827 *± *0.031	0.805 *± *0.009	0.771 *± *0.008	0.789 *± *0.007	**0.834 ***± ***0.020**
4%	0.833 *± *0.021	0.813 *± *0.026	0.805 *± *0.016	0.800 *± *0.006	**0.845 ***± ***0.027**
5%	**0.843 ***± ***0.008**	0.802 *± *0.018	0.804 *± *0.024	0.803 *± *0.016	**0.843 ***± ***0.012**
6%	0.846 *± *0.004	0.807 *± *0.023	0.790 *± *0.017	0.818 *± *0.003	**0.849 ***± ***0.013**
7%	0.846 *± *0.002	0.827 *± *0.018	0.812 *± *0.019	0.845 *± *0.005	**0.868 ***± ***0.013**
8%	0.852 *± *0.002	0.813 *± *0.011	0.817 *± *0.030	0.845 *± *0.002	**0.860 ***± ***0.008**
9%	0.857 *± *0.020	0.831 *± *0.014	0.826 *± *0.022	0.853 *± *0.004	**0.872 ***± ***0.011**
10%	0.858 *± *0.017	0.831 *± *0.014	0.846 *± *0.012	0.855 *± *0.007	**0.874 ***± ***0.006**

We report the average accuracy and standard deviation of the comparison methods over 10 runs. The numbers in boldface (on each row of the tables) indicate the best results for each label ratio over the methods. From Table 1, we observe that EGM-SMCC using multiple latent graphs is able to achieve better performance against the GM-SMCC method using a single latent graph. A reasonable explanation for this finding is that the different latent graphs have complementary relationship for prediction. These latent graphs are derived from different sources. When complementary models learned from these latent graphs are combined in an ensemble, correct decisions are amplified by the aggregation process. The performance of an ensemble learner is highly dependent on two factors: one is the accuracy of each component learner; the other is the diversity among these components. Examining the results in Table 1 shows that the overall performances of the GM-SMCC models generated from different graphs are reasonably well. This result indicates that each latent graph provides prediction knowledge from a specific aspect, and their combination leads to a more robust prediction.

### Results on protein function prediction

We also conduct experiments for problem (2) of KDD Cup 2001, i.e., the multi-label protein function prediction problem. We set *α *and *β *to be non-zero by considering the network information and label correlation simultaneously. We compare the proposed algorithms with baseline classifiers: SVM, wvRN+RN, ICA, semi-ICA and ICML. SVM, wvRN+RN, ICA and semi-ICA are single-label classifiers. For these methods, we decompose the multi-label problem into a set of *K *binary classification problems using one-against-all strategy, and train independent classifier for each single-label problem. This approach is known as the binary relevance (BR) method [40]. The predictions for all *K *binary classification problems are combined to make the final prediction.

**Figure 3:**
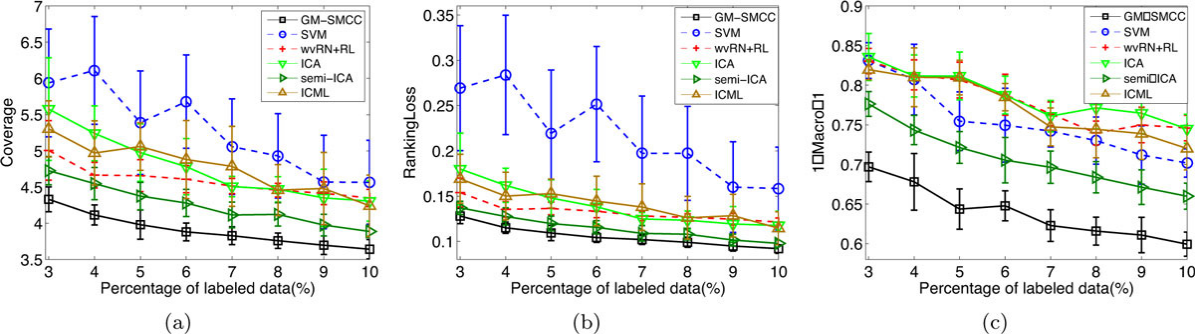
**The performance of the algorithms with varying percentages of labeled data on problem (2) of KDD Cup 2001**. (a)Coverage; (b)RankingLoss; (c)1-Macro-F1.

We compare the performance of our proposed GM-SMCC approach and other baseline algorithms with varying percentages of labeled data from 3% to 10%. For each percentage, we execute each algorithm 10 times by randomly selecting the label/unlabel data split from the dataset. Then we report average results as well as standard deviation of each compared algorithms over 10 runs. The result is shown in Figure 3. In order to keep consistency with the *Coverage *and *RankingLoss *evaluation metrics, we use 1-*MacroF1 *instead of *MacroF1*. Thus, the smaller the value of the metric, the better the performance of the algorithm. We see from Figure 3 that GM-SMCC (the black line) has the best performance (lies under the other curves) across all evaluation metrics and label ratios. Semi-ICA is the second best method. In the comparison, SVM performs poor in terms of *Coverage*. On the other hand, wvRN+RL, ICML and ICA perform poor in terms of *MacroF1*. Recent studies [41] have shown that one multi-label learning algorithm rarely outperforms another algorithm on all criteria because the evaluation measures used in the experiments assess the learning performance from different aspects. In the experiments, we find that GM-SMCC consistently out-performs other algorithms across all label ratios. On average, ICAM achieves *Coverage *improvement of 0.35 (GM-SMCC:3.90 versus semiICA:4.25), *RankingLoss *improvement of 0.01 (GM-SMCC:0.104 versus semiICA:0.114), and 1-MacroF1 improvement of 0.068 (GM-SMCC:0.640 versus semiICA:0.708) against the second best method. This result indicates that the proposed GM-SMCC algorithm is effective for the multi-label protein function prediction task.

Similar to the experiments for protein localization prediction, we also conduct experiments to examine the effect of the proposed EGM-SMCC method (integrating multiple latent graphs) for enhancing the prediction performance against the GM-SMCC method using a single latent graph. GM-SMCC-1, GM-SMCC-2 and GM-SMCC-3 denote the single-graph model using (*E*^(1)^), (*E*^(2)^) and (*E*^(3)^), respectively. GM-SMCC-mean denotes the single-graph model using a latent graph constructed by averaging the weighing values of *E*^(1)^, *E*^(2) ^and *E*^(3)^.

**Figure 4:**
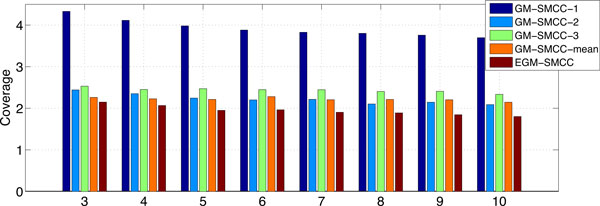
**The *Coverage *of EGM-SMCC and GM-SMCC with various latent graphs: GM-SMCC-1 (PPI latent graph), GM-SMCC-2 (random walk latent graph), GM-SMCC-3 (prediction similarity graph), GM-SMCC-mean (a single graph model averages the weighting values of *E*^(1) ^, *E*^(2) ^and *E*^(3) ^) with respect to different percentages of labeled data (%) for the problem (2) of KDD Cup 2001**.

**Figure 5:**
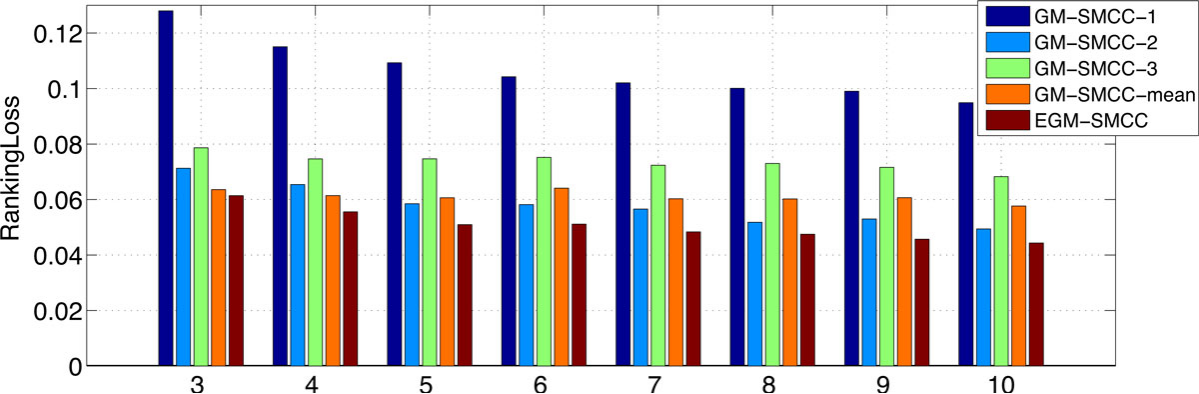
**Same as Figure 4, but for *RankingLoss *evaluation metric**.

We compare GM-SMCC and EGM-SMCC with respect to different percentages of labeled data from 3% to 10%. For brevity, we just report *Coverage *and *RankingLoss*. The results are given in Figure 4 and 5. The percentage of labeled data is illustrated on the horizontal axis. According to the figures, we can see that EGM-SMCC consistently outperforms the GM-SMCC algorithms using a single latent graph because more information are utilized. This result demonstrates the effectiveness of our proposed EGM-SMCC method for multi-label protein function prediction.

### Convergence study

**Figure 6:**
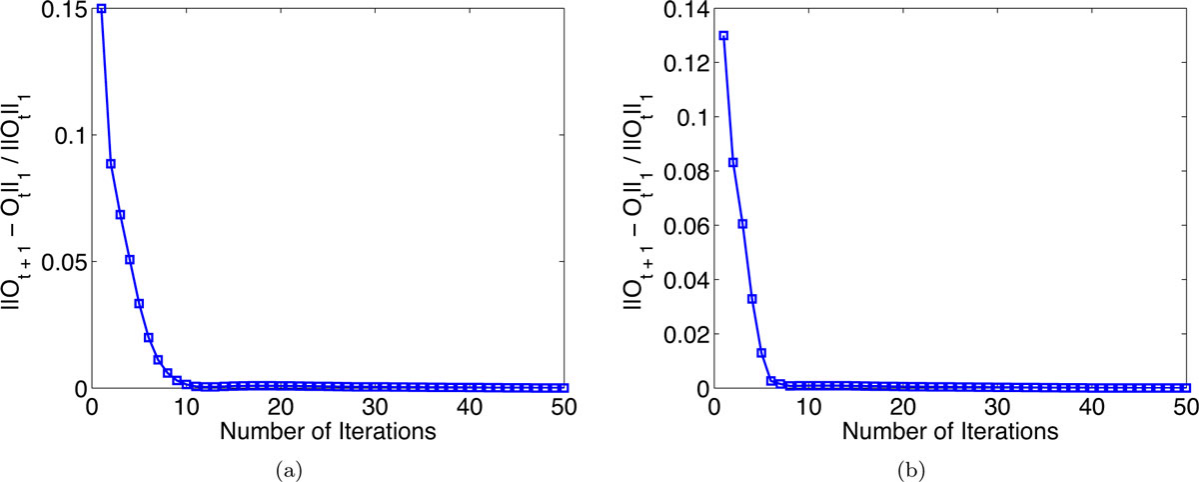
**The convergence curves of the proposed method**. (a) Convergence curve on the problem (1); (b) convergence curve on the problem (2).

The objective function  O in Eq. (4) is optimized for classification prediction. Here, we investigate how fast the algorithm converges. Figures 6(a) and 6(b) show the convergence curves of the proposed algorithm on the problem (1) and (2) (at 5% label ratio), respectively. The *x*-axis is the number of iteration number in the process of optimizing the objective value O and the *y*-axis is the value of successively computed objective value ||O(t+1)-O(t)||/||O(t)||. We see that the algorithm converge within 10 iterations. The required computational time for problems (1) and (2) are 10.5 seconds and 10.3 seconds using our MATLAB implementation, respectively.

### Parameter sensitivity

In our proposed GM-SMCC method, the regularization parameters *α *and *β *quantify the importance of the network regularizer and label regularizer in the objective function (4). These parameters also determine the learning setting. Our framework is formulated in single-label collective classification learning by considering *α ≠ *0 and *β *= 0, i.e., we solve single label learning problem for the problem (1). On the other hand, our framework is formulated in multi-label collective classification learning when *α ≠ *0 and *β *≠ 0, i.e., we consider the label correlation in the learning process for the problem (2).

**Figure 7:**
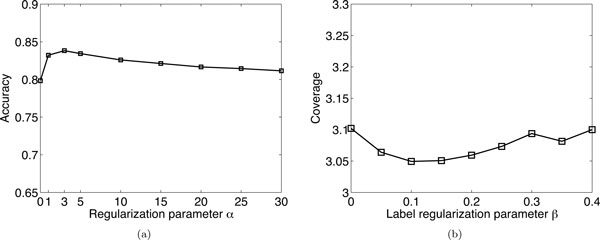
**The performance of the proposed method with different parameter values on the KDD Cup 2001 dataset**. (a) The accuracy of different *α *values on problem (1); (b) the *Coverage *of different *β *values on problem (2).

We examine the parametric sensitivity of our GM-SMCC approach with respect to parameter *α *by fixing *β *= 0 and varying *α *on problem (1). Figure 7(a) illustrates the accuracy of GM-SMCC with different *α *values from 0 to 30 on the protein localization prediction task using 5% label ratio. When *α *= 0 the accuracy is low, since no network information is used in this case. This also provides evidence of the advantages of the network regularization in the proposed method. When *α *becomes large, the accuracy increases. The plateau in the accuracy curve from 1 to 30 shows that the proposed GM-SMCC achieves fairly stable performance with different value of *α*. It implies that the method is robust when a different value of *α *is selected. We find that GM-SMCC presents good classification performance when *α *= 3.

Next, we fix *α *= 3 and vary *β *from 0 to 0.4 on problem (2) using 5% label ratio. The result is given in Figure 7(b). We observe that when *β *= 0 or *β *= 0.4, the performance is poor. It is evident that the smallest *Coverage *is achieved at *β *= 0.1. Therefore, we set *α *= 3 and *β *= 0.1 in all the comparisons.

### Interaction relations

Our proposed method using the objective function in Eq. (4) is capable characterizing the interaction relations among the genes code for proteins, and these proteins tend to localize in various parts of cells in order to perform crucial functions. We construct an extended graph data set $G^{\prime }=\left(X^{\prime },E^{\prime }\right)$ for the KDD Cup 2001 data, where $E^{\prime }$ is the known interactions among the proteins and $X^{\prime }$ is the feature set of the proteins. Each $x_{i}^{\prime }\notin X^{\prime }$ is an extended feature vector for the *i*-th protein/gene by integrating its attribute features, localization and functional labels together as follows: $x_{i}^{\prime }=\left(x_{i},Y_{i}^{l},Y_{i}^{f}\right)$, where *x_i _*is the attribute vector, $Y_{i}^{l}=\left[Y_{i1}^{l},Y_{i2}^{l}\right]\in {\left\{0,1\right\}}^{2}$ and $Y_{i}^{f}=\left[Y_{i1}^{f},\cdots \;,Y_{iK}^{f}\right]\in {\left\{0,1\right\}}^{K}$ are the localization label features and function label features with respect to *i*th instance. Given a new instance $\hat{x}$, the interaction between $\hat{x}$ and $x_{i}^{\prime }\in X^{\prime }$ is estimated by the cosine similarity between their conditional probability vectors obtained from the proposed method. The resulting similarity ranges from 0 to 1, with 0 indicating two instances are independent, and 1 indicating two instances are highly interrelated. We apply the cosine similarity measure to evaluate the interaction relations of 5 randomly selected genes (G238510, G234935, G235158, G237021, G234980) to other genes in the KDD Cup 2001 dataset. Table 2 shows the interesting interrelations discovered by previous studies with respect to the evaluated genes. In general, we can see that these interrelated genes tend to have large similarity values. This shows the advantages of using our proposed method to detect the interactions. Biologists can use the method to identify related genes and to further investigate their interactions.

**Table 2:** Selected interrelated genes and their similarity computed by the proposed GM-SMCC method.

GeneID	GeneID	Similarity
G238510	G239467	0.99706
G238510	G239178	0.95597
G238510	G235250	0.8347
G234935	G234445	0.9178
G234935	G239966	0.92039
G234935	G235763	0.95516
G234935	G235329	0.95938
G235158	G234735	0.98431
G235158	G234074	0.9788
G235158	G234177	0.90675
G235158	G235216	0.96184
G237021	G234486	0.85557
G237021	G234065	0.88554
G237021	G239804	0.96585
G237021	G239266	0.92513
G234980	G235439	0.98653
G234980	G235231	0.99427
G234980	G234914	0.99755
G234980	G235780	0.96058

## Conclusion

In this paper, we first propose GM-SMCC, an effective and novel semi-supervised multi-label collective classification based method for predicting functional properties of proteins. GM-SMCC is designed with the use of pLSA generative model with a network regularizer and label regularizer, which exploit the network linkages and label correlations effectively to compute the label probability distribution for prediction. Then, we extend it in an ensemble manner and develop the EGM-SMCC approach to exploit various kinds of latent linkages in constructing latent graphs to further improve the prediction performance. Experimental results on two tasks of KDD Cup 2001 (the localization prediction task and the protein function prediction task) consistently demonstrate the effectiveness of the proposed methods. The performances of the proposed methods are shown to be better than that of state-of-the-art algorithms, including SVM, wvRN+RL, and three variants of ICA. In future, we will extend our proposed method to handle heterogeneous biological networks.

## Competing interests

The authors declare that they have no competing interests.

## Authors' contributions

Q. Wu participated in designing the algorithm and drafted the manuscript. Y. Ye, S.S. Ho and S. Zhou revised and finalized the paper. All authors read and approved the final manuscript.
